# Supplementary figures and images for: Nature Forest Reserves in Tanzania and their importance for conservation
Source: PLoS One. 2024 Feb 5;19(2):e0281408. doi: 10.1371/journal.pone.0281408 (PMC10843475; doi:10.1371/journal.pone.0281408)

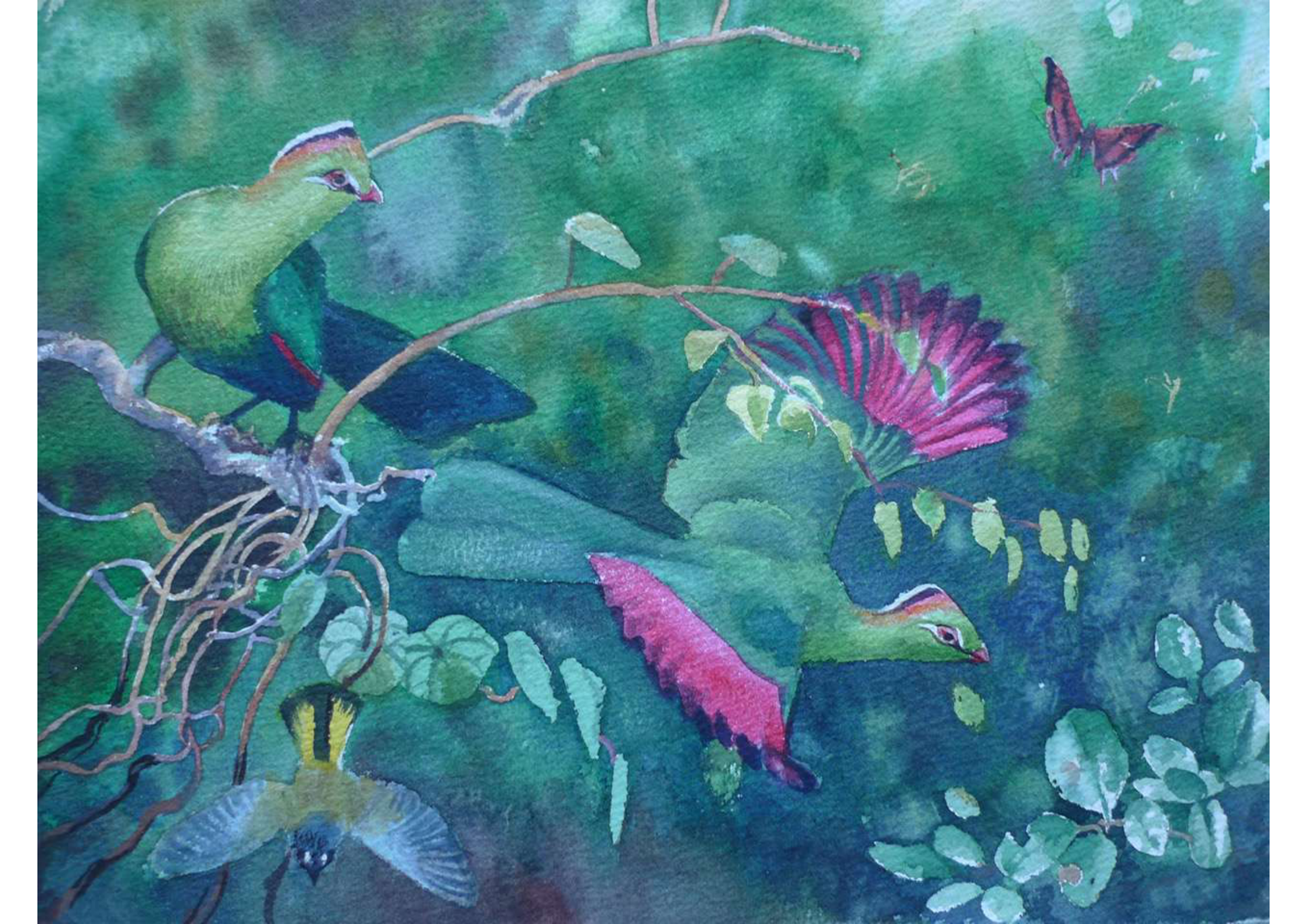

Supplement: S1 Fig — Painting 2: The narrowly endemic Udzungwa forest partridge (Xenoperdix udzungwensis), and grey-faced sengi (Rhynchocyon udzungwensis) encountering each other on the forest floor in the Kilombero Nature Reserve in the Udzungwa Mountains within the Eastern Arc mountains region. (ZIP) [file pone.0281408.s001.zip › Figure S1.tif]

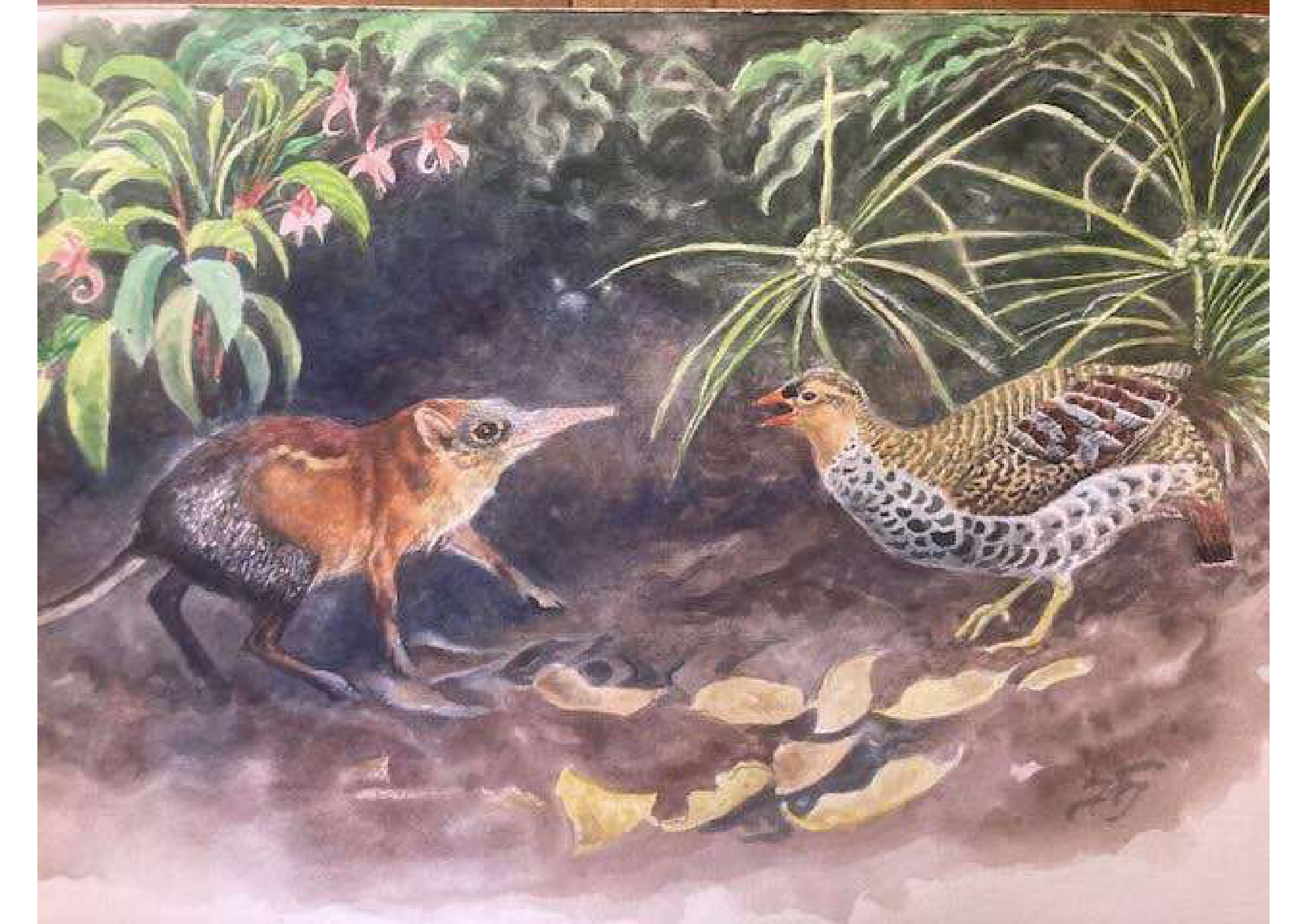

Supplement: S1 Fig — Painting 2: The narrowly endemic Udzungwa forest partridge (Xenoperdix udzungwensis), and grey-faced sengi (Rhynchocyon udzungwensis) encountering each other on the forest floor in the Kilombero Nature Reserve in the Udzungwa Mountains within the Eastern Arc mountains region. (ZIP) [file pone.0281408.s001.zip › Figure S1_1.tif]

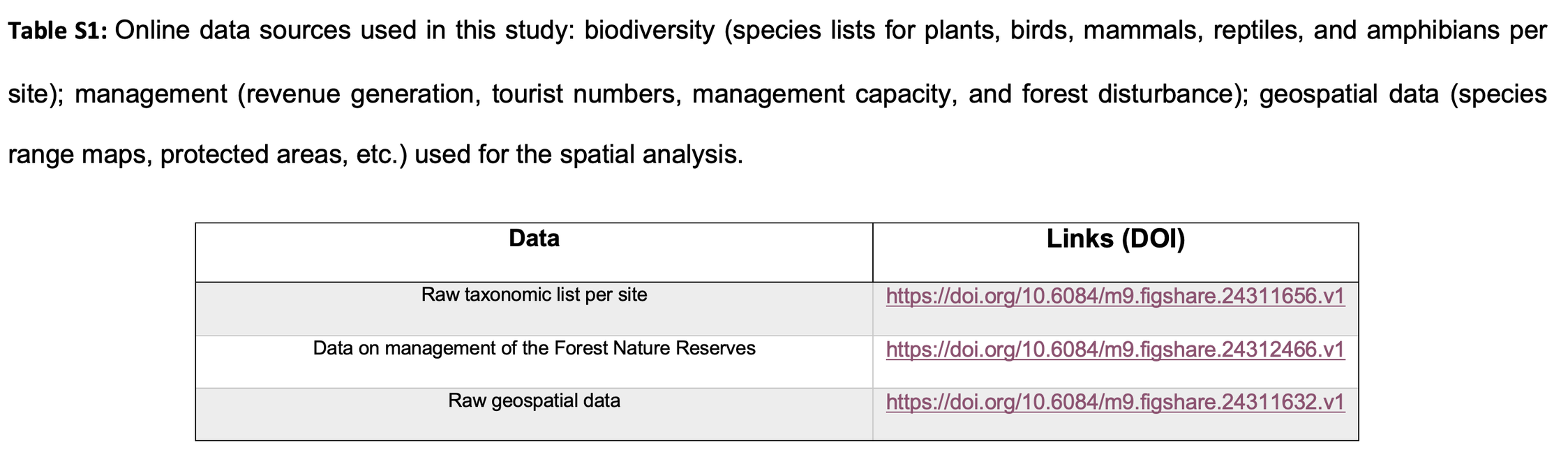

Supplement: S1 Table — (TIF) [file pone.0281408.s002.tif]

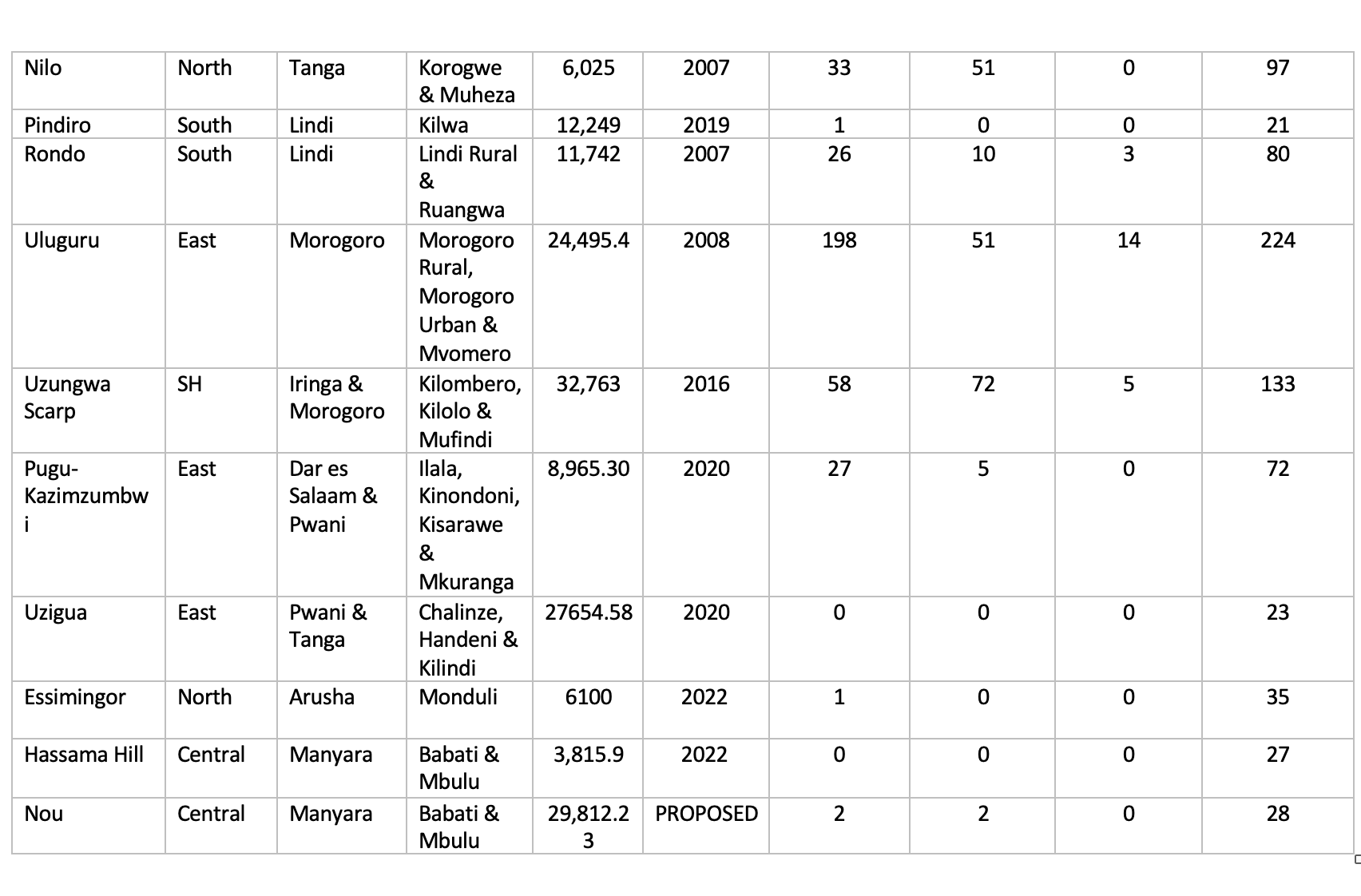

Supplement: S2 Table — (ZIP) [file pone.0281408.s003.zip › Table S2/Table-S2-2.tif]

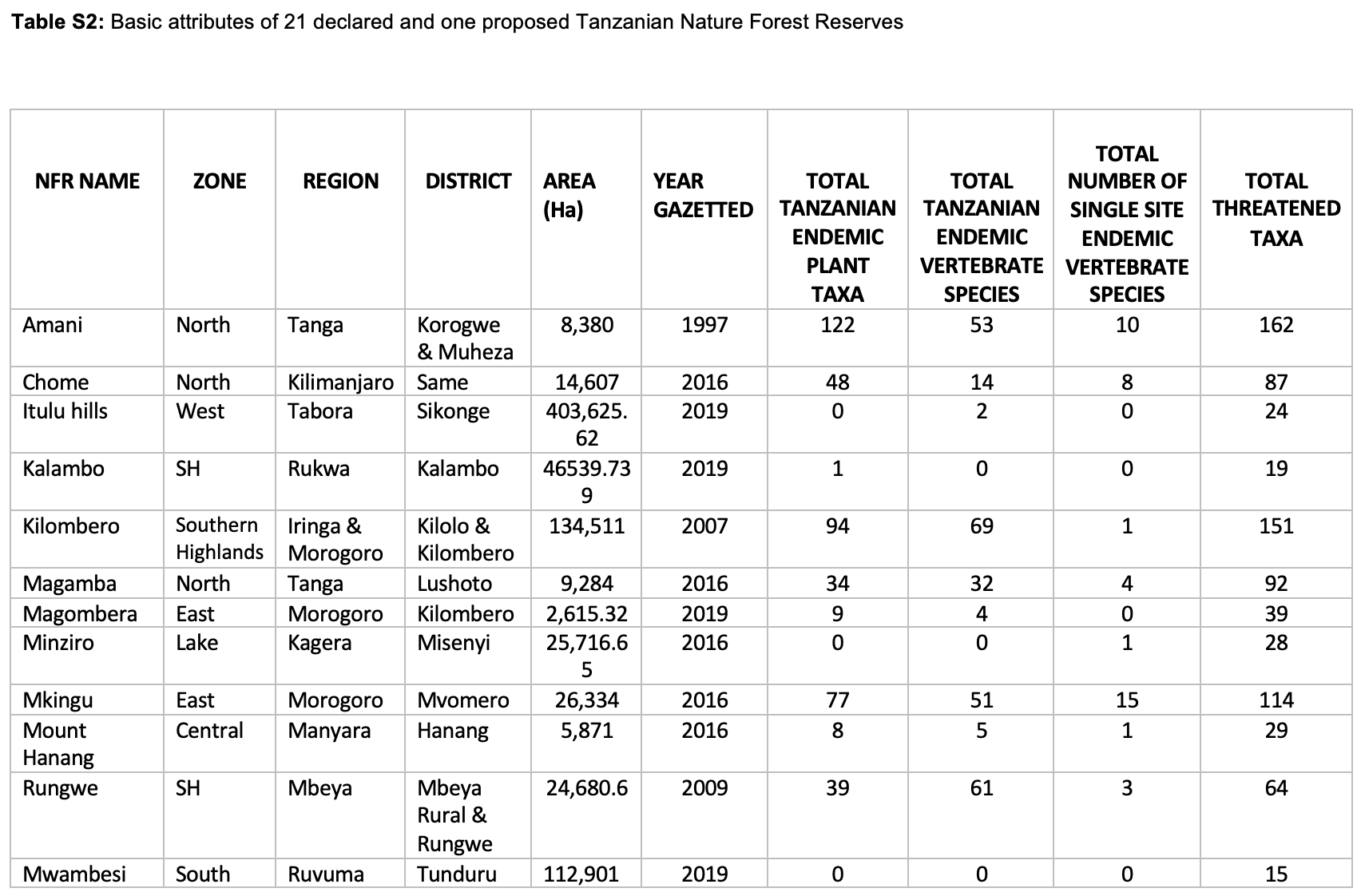

Supplement: S2 Table — (ZIP) [file pone.0281408.s003.zip › Table S2/Table-S2-1-.tif]
