# Supplementary material for: Nature Forest Reserves in Tanzania and their importance for conservation
Source: PLoS One. 2024 Feb 5;19(2):e0281408. doi: 10.1371/journal.pone.0281408 (PMC10843475; doi:10.1371/journal.pone.0281408)
Supplement: Attachment — Submitted filename: Manuscript_final.docx [file pone.0281408.s004.docx]

**Nature Forest Reserves in Tanzania and their importance for conservation**

**Short title: Forest Reserves in Tanzania**

**Authors**: Claire Ract^1,^ Neil D. Burgess^1,2^, Lars Dinesen^1^, Peter Sumbi^3^, Isaac Malugu^4^, Julia Latham^5^, Lucy Anderson^6^, Roy E. Gereau^7^, Marcelo Gonçalves de Lima^8^, Amina Akida^9^, Evarist Nashanda^9^, Gertrude Lyatuu^10^, Phil Platts^11, 12^ and Francesco Rovero^13^

**Dedication**: To those who put their heart and soul into creating a network of Tanzanian Nature Reserves and died too early along the way: Alan Rodgers, John Mejissa, Corodius Sawe, Gerald Kamwenda, Peter Sumbi

**Addresses**

^1^ Centre for Macroecology Evolution and Climate (CMEC), GLOBE Institute, University of Copenhagen, Universitstsparken 15, Copenhagen, Denmark

^2^ United Nations Environment Programme World Conservation Monitoring Centre (UNEP-WCMC), 219 Huntington Road, Cambridge, UK

^3^ Dar es Salaam, Tanzania (Deceased)

^4^ Environment and Forest Certification Limited (EFC), Dar es Salaam, Tanzania

^5^ Consultant, Conservation Insight, UK.

^6^ Consultant, Conservation Insight, UK.

^7^ Missouri Botanical Garden, St. Louis, MO, USA

^8^ Cambridge Wildlife Trust, Cambridge

^9^ TFS, Mpingo House, Dar es Salaam, Tanzania

^10^ UNDP, UN House, Masaki, Dar es Salaam, Tanzania

^11^ Department of Environment and Geography, University of York, York, YO10 5NG, UK

^12^ BeZero Carbon Ltd, Discovery House, London, EC1Y 8QE

^13^ Department of Biology, University of Florence, Sesto Fiorentino, Italy. MUSE – Museo delle Scienze, Trento, Italy.

*Corresponding author

E-mail: [ractclaire@gmail.com](mailto:ractclaire@gmail.com)

**Key words:** Protected Areas. Biodiversity. Ecotourism. Africa

**Abstract**.  Since 1997 Tanzania has undertaken a process to identify and declare a network of Nature Forest Reserves (NFRs) with high biodiversity values, from within its existing portfolio of national Forest Reserves and the addition of ten NFRs declared since 2015. The current network of 19 NFRs covered 918,212 hectares in 2020 (with an additional three reserves covering 34,862 hectares in the process of being declared). The NFRs now cover a wider range of Tanzanian habitat types, including the main forest types, wet, seasonal, and dry, as well as wetlands and grasslands. This has more than doubled the area of vertebrate species ranges covered by the NFR network. Declared and proposed reserves now contain at least 178 of Tanzania’s 242 endemic vertebrate species, of which at least 50% are threatened with extinction, and 553 Tanzanian endemic plant taxa (species, subspecies, and varieties), of which 61.3% are threatened. NFRs also support 41 single site endemic vertebrate species and 76 single site endemic plant taxa. Time series data show that NFR management effectiveness is increasing, especially where donor funds are available.  Tourism, diversified revenue generation and investment schemes are required to create a network of economically self-sustaining NFRs able to conserve critical biodiversity values. Improved management and investment have reduced some threats in recent years, but ongoing challenges include illegal logging, charcoal production, firewood, pole-cutting, hunting, fire, wildlife trade, and the unpredictable impacts of climate change.

**Introduction**

Protected areas are essential for conserving biodiversity and storing carbon and are an important buffer against climate change [1,2]. The International Union for Conservation of Nature (IUCN) defines a Protected Area (PA) as “An area of land and/or sea especially dedicated to the protection and maintenance of biological diversity, and of natural and associated cultural resources, and managed through legal or other effective means” [1].

The creation of protected forest areas in Tanzania has a long history stretching back to the German colonial period in the late 1800s. This ‘forest reserve’ network has expanded over time, first during the German and subsequent British colonial periods, and then since Tanzanian independence in 1961 [3, 4, 5, 6]. Management aims include reserves established for production from natural forests (timber and charcoal), protection of natural forests (water catchment reserves and for the prevention of landslides and erosion), and plantation forestry using exotic species.

After the implementation of the ‘new’ Forest Policy in 1998 [7] and the Forest Act in 2002 [8], forest reserves were designated specifically for the preservation of their biodiversity and habitats, and human activities were restricted [4, 5]. Under this new legislative framework, Nature Forest Reserves (NFRs) are recognised as forest areas of particularly high importance for globally unique biodiversity and managed in most cases with strong protection. The first phase of declaring NFRs was in the Eastern Arc Mountains ecoregion [9] during the period 1997-2009, starting with Amani Nature Reserve in the East Usambara Mountains in 1997.

Over the past 25 years, the Tanzanian government has continued to identify and upgrade other biologically important reserves to become NFRs. These sites initially fell under the ownership and management of the Forestry and Beekeeping Division of the Ministry of Natural Resources and Tourism, and (since 2005) the Tanzania Forest Services (TFS) Agency. After the first phase of declaring NFRs, the network expanded to cover all the different forest types in the country (including the Coastal forests, the Northern Volcanic, the Southern Highlands, Guineo-Congolian, Miombo and Miombo-Acacia forests). The current network of NFRs in Tanzania contains 19 reserves, with an additional three reserves in the process of being declared (Figure 1).

We describe and assess Tanzania’s NFR network for the first time by: (i) evaluating the development of the NFR network in Tanzania and assessing its coverage of Tanzanian biodiversity values; (ii) assessing the effectiveness of NFRs and whether management resources have been deployed to the most important sites to achieve conservation goals; and (iii) assessing the coverage by the NFR network and other protected areas across Tanzania of endemic and rare species. We provide current insights into the management challenges in the NFR and how these might be addressed in the future.

*Fig 1*: Map of Tanzania showing Nature Forest Reserves (NFRs, red), as well as Forest Reserves (green) including three in the process of being declared NFRs (italicized names), and other kinds of protected areas (shaded).

**Methods**

**Data collection**

Between September 2019 and November 2021, we collated available published and unpublished data on the Tanzanian NFRs (summarised in Table 1). Data on 19 declared NFRs and 3 proposed NFRs up to 2020 included declaration details, biological features, management information, and threats. Management data on the last five NFRs were not available as they were either just declared (Pugu-Kazimzumbwi and Uzigua) or proposed (Essimingor, Hassama Hill, and Nou).

Detailed lists of vertebrate species and plant taxa in the reserves were built upon existing lists for the Eastern Arc Mountains [10, 11, 12, 6], and for coastal forests [13]. Unpublished lists of plant taxa in each Nature Reserve, collated by Roy Gereau of the Missouri Botanical Garden in 2020, were augmented with records from the GBIF portal (accessed in July 2020), and data compiled for other sites using GIS overlay analyses. The designation “taxa” will be used throughout the text to mean “terminal taxa” for plants including species, subspecies, and varieties. However, the unit of analysis for vertebrates is species only. Vertebrate species distribution data were compiled from existing GIS data: birds [14], amphibians and mammals [15], and reptiles [16]. Species occurrences were determined within the NFR shapefiles provided by the Word Database on Protected Areas, maintained by the United Nations Environment Programme - World Conservation Monitoring Centre (UNEP-WCMC). Additional data sources included published and unpublished reports such as forest management plans and biodiversity survey reports, as well as data from unpublished baseline, mid-term, and end-line NFR surveys undertaken as part of a Global Environment Facility (GEF) -funded project “*Enhancing the Forest Nature Reserves Network for Biodiversity Conservation in Tanzania’*. Data on the taxa occurring in each of the 19 NFRs and 3 proposed NFRs were categorised as endemic (both endemic to Tanzania or to the NFR itself) and threatened with extinction according to IUCN Red List categories: Critically Endangered (CR), Endangered (EN), and Vulnerable (VU).

Declaration date and area for each NFR were gathered from Tanzanian government records. Assessment of NFR management effectiveness was derived from the Management Effectiveness Tracking Tool (METT) developed by the World Commission on Protected Areas [17]. METT assessments were applied four times, first at the start of the GEF project in 2015; then at project mid-point in 2017; and finally at project endpoint in 2019. A recent assessment of the 17 NFR sites for which there were data was completed in 2021 using the newest methodology METT 4.0. High METT scores (range = ? to ?) indicate better management effectiveness [18, 19]. Data on income generated and tourism trends were collated from the managers of each NFR, reserve management plans, and Project Implementation Reports (PIR) to the GEF through the UNDP Tanzania country office. In 2019, compiled data were checked by managers of all 17 NFRs and the staff of UNDP-Tanzania and the Tanzania Forest Service [20]. To assess management effectiveness further, data on forest disturbance were collected for 4 NFRs (Amani, Kilombero, Mkingu, and Uluguru) where baseline (2001 - pre NFR creation) and more recent (2019) data exist. Data on disturbance were gathered from 10 m wide by 600 m long transects within the forest (total length surveyed of 3.3km; [21]). Data on live, naturally dead, or cut stems of trees were collected. The aim of repeating disturbance transects was to determine whether the declaration of NFR status has reduced habitat degradation.

**Data analysis**

We assessed the biodiversity value of each reserve from the number of both endemic and threatened taxa in each reserve. Reserves were then ranked in chronological order of declaration to visualise the change in biodiversity value of the forest network over time. The correlation between the number of taxa and the date of declaration was tested using the non-parametric Spearman’s rank correlation coefficient.

The Management Effectiveness Tracking Tool (METT; [17]) was used to assess and compare the effectiveness of management over time in each NFR, alongside tourist numbers, income generated, management capacity, and forest disturbance. Possible correlations among the covariates were tested using the Spearman rank correlation test in R.

To determine the change in endemic vertebrate species coverage over time within the growing NFR network, we calculated the percent of endemic species ranges inside and outside the reserves and determined the presence of gap(s) or poorly covered species ranges. Species distribution data from the IUCN Red List for amphibians, reptiles, birds, and mammals were combined with NFR location data to establish species coverage by the NFR network. We grouped NFRs according to the date they were established, resulting in eight groups/time frames (as several reserves were declared the same year). We chose to focus only on species endemic to Tanzania. For each endemic species, the percent of its range covered by the sites was determined and classified into different categories: (1) gap species: 0% of an endemic species’ range covered by the sites, (2) poorly covered species: between 0-2% of an endemic species’ range covered by the sites, (3) 2-5% of an endemic species’ range covered by the sites, (4) 6-10% of an endemic species’ range covered by the sites, (5) between 11-20%, (6) between 21-50%, and (7) more than 50% of an endemic species’ range covered by the sites. The proportion of endemic species was calculated in each of the categories.

We also created species richness maps to visualise the gap analysis. Only the ranges of ‘endemic gap’ species and ‘poorly covered endemic’ species were used. We used the World Database on Protected Areas (accessed in April 2022) to perform a further gap analysis to determine if there are other types of protected areas, in addition to NFRs, that overlap with the gap/poorly covered species still present once all 22 NFRs were included in the analysis. We combined all types of protected areas occurring in Tanzania such as National Parks, Nature Reserves, Game Reserves, Forest Reserves, Conservation Areas, Village Land Forest Reserves, and Game Controlled Areas. We determined the percent of species range covered by the different types of protected areas and computed the proportion of species in each of classification categories. Analyses used the statistical software R (version 4.0.5) [22] and the spatial Geographic Information System QGIS (version 3.16.14).

| Data | Links |
| --- | --- |
| Raw taxonomic list per site | [biodiversity data](https://drive.google.com/drive/folders/1zYLaYld__pC4Z0vCjXKMbr622c6QzyJe) |
| Data on management of the sites | [management data](https://drive.google.com/drive/folders/1mPQnr-ANuWHABGOr2EBarAB5ZKnk_ASR) |
| Raw geospatial data | [spatial data](https://drive.google.com/drive/folders/149BjTwk3N77pgGhHDaC2ebO8loquTI57) |

**Table 1:** Online data sources used in this study: biodiversity (species lists for plants, birds, mammals, reptiles, and amphibians per site); management (revenue generation, tourist numbers, management capacity, and forest disturbance); geospatial data (species range maps, protected areas, etc.) used for the spatial analysis.

**Results**

**Development of the NFR network over time and coverage of Tanzanian biodiversity values**

Older NFR sites supported a larger number of threatened taxa than recently declared sites (Figure 2A and Table 3). Amani, Kilombero, and Uluguru NFRs had the highest number of threatened taxa, followed by Nilo, Chome, Mkingu, Uzungwa Scarp, Rondo, and Magamba. Many of the threatened species were also endemic to Tanzania (see Figure 2B, Amani to Mangula). As expected, as new reserves were added to the network there was a gradual increase in the number taxa in the NFR network, but this was found to asymptote for all reserves declared since 2019 (Figure 2C). The cumulative numbers of endemic taxa was correlated with total taxa and date of declaration of the reserves (Figure 2, r_s_ = 0.97, p < 0.001 for all taxon groups). This significant increase is also found in each taxon group separately (r_s_= 0.95, p < 0.001 for each taxon group). We found no significant correlation between the year of establishment and the reserve area (p = 0.8755).

*Fig 2:* The change in importance of the network of NFRs for Tanzanian endemic and globally threatened plant since 1977. A: Number of threatened taxa in the NFRs. B: Cumulative number of endemic taxa. C: Cumulative number of total taxa (including endemic, threatened, and other taxa). Reserves are ordered according to the year of their designation (the last three reserves are proposed with no year of designation).

**Effectiveness of NFR management in achieving conservation goals**

The effectiveness of management of the NFR network increased over time. For the 11 NFRs present in 2015 the METT percent was 55%, 69% in 2017 for 12 NFRs, 69% in 2019 for 17 NFRs assessed, and in 2021 increased to a mean score of 86.8% for the 17 NFRs evaluated (Figure 3A). However, the Spearman correlation test did not find any significant relationship between the date and the average final METT scores across the reserves (Figure 3A). NFRs rich in endemic taxa had better management effectiveness (Figure 3B), but the relationship was not significant (Spearman correlation test, effect size=0.03, p = 0.46) (Figure 3B). Total tourist numbers (mainly international visitors) increased steadily in most NFRs, from 1698 tourists in 2016 to 4097 tourists in 2019 (Figure 3C; r_s_ = 0.95, all p values < 0.0123, N = 17). However, income generated (US dollars fixed across the four years measured) decreased slightly from 2017 to 2019 (Figure 3: D). Furthermore, no significant relationship was found between the average income generated and the four years studied, except between 2017 and 2021 (Spearman correlation test, effect size= 0.8095, p value = 0.02178, N = 17) (Figure 3: D). Management capacity, including the number buildings, transport, and equipment present in each NFR increased between 2015 and 2019, but the number of staff and rangers declined in 2019 (Figure 3: E). Only large equipment (corresponding to the number of computers, photocopier scanner, printers, GPS units, solar batteries, and hard drives) increased between 2015 and 2019.

*Fig 3:* Changes in management from 2015-2021 for: A) mean METT score across the sites; B) relationship between average METT score and biodiversity value ranked in ascending order (linear trend line not significant); C) annual number of tourists in all sites (Table S1 for full data); D) average total revenue generated in the reserves (US dollars); E) sum of the management capacity in 2015 and 2019 comprising staffing (staff and rangers), buildings (offices and ranger posts), transport (vehicles), and small equipment.

Analysis of forest disturbance transect data from four NFRs in 2001 (before these NFRs were declared - except for Amani) and 2019 shows that numbers of cut trees and cut poles per hectare have declined since the reserves were declared (except for Uluguru and Kilombero NFRs) (Figure 4). The number of live trees was stable (in Uluguru NFR) or decreased (in Kilombero and Mkingu NFRs), while the number of live poles increased (except for Kilombero NFR).

*Fig 4:* Changes in tree harvesting: A) living trees (>20 cm dbh), B) cut trees, C) living poles (<20 cm dbh), and D) cut poles in Amani, Kilombero, Uluguru, and Mkingu Nature Forest Reserves before reserve declaration and in February 2019.

**NFR network coverage of endemic and threatened species**

As the number of sites added to the NFR network increased, the number of endemic vertebrate species in the reserve network increased and gap species declined. For Tanzanian endemic bird species, all previous gap species were covered after the addition of the last NFR sites to the network (Figure 5: A). However, the proportion of poorly covered endemic bird species (between 0-2% of their range covered) increased and was the highest across all of the endemic vertebrate groups. For mammals, gap species declined to 5% when all sites were included in the analysis (Figure 5: B). In the full NFR network, the proportion (??%) of endemic amphibian species with more than 50% of their range protected was higher than other taxa, with more than 25% of endemic amphibian species ranges partially covered by the NFRs (Figure 5: C). Amphibians presented the highest proportion of gap species for all sites combined (Figure 5: C). As for the other vertebrate groups, the proportion of endemic reptile gap species declined to 10% as new NFR sites were added to the network (Figure 5: D).

*Fig 5*: Proportion of Tanzanian endemic vertebrate species ranges covered by NFRs over time. Years when the reserves were gazetted are in parentheses and the number of reserves declared in each time frame is indicated after “Amani”. The light brown bars refer to the endemic gap species, where 0 percent of their range is covered. Other colours show the percentage of endemic species range covered by reserves.

Richness maps of all endemic gap species show, not surprisingly, a decline in the proportion of gap species as more reserves were added to the network. However, there is a gap in NFR network coverage for endemic reptile species in south-eastern Tanzania (Rondo and Pindiro regions) and in some central regions (Figure 6).

*Fig 6*: Coverage of the range of Tanzanian endemic ‘gap’ species (panels A, B, C) and poorly covered endemic species (panels D, E, F). Panels A and D show endemic species coverage in 1997, when the first reserve was added to the network. Panels B and E show the endemic species coverage in 2017, with 9 reserves in the network. Panels C and F represent the species coverage in 2022, with 19 NFRs and 3 proposed NFRs included in the network. The orange coverage range displays endemic mammal species, blue reptile species, green bird species, and light pink amphibian species. The NFRs are indicated as black polygons.

Including all types of protected areas present in Tanzania, the proportion of gap and poorly covered species is much lower compared to only NFRs (Figure 7). No poorly covered species were found in all protected areas, compared to 30% of poorly covered bird species in the NFRs network (Table 2). Almost 7% of endemic amphibian species remain unprotected, compared with approximately 11% in NFRs (Table 2).

*Fig 7:* Coverage of the ranges of gap and poorly covered endemic Tanzanian vertebrate species in the protected area network in Tanzania. Orange shows location of endemic mammal species, blue - reptiles, and green represents endemic amphibian species. There are no gap species of birds. Protected areas are indicated as grey polygons.

**Table 2:** Comparison of the proportion of gap and poorly covered endemic amphibian, reptile, bird, and mammal species, when all protected areas are included compared to only the NFRs

|  | All types of protected areas | | All NFRs | |
| --- | --- | --- | --- | --- |
|  | Gap species | Poorly covered species | Gap species | Poorly covered species |
| Amphibian species | 6,52 | 0 | 10,77 | 13,85 |
| Reptile species | 6,52 | 6,52 | 7,94 | 22,22 |
| Bird species | 0 | 0 | 0 | 30,769 |
| Mammal species | 2,17 | 0 | 5,26 | 10,53 |

**Discussion**

*How well do NFRs capture Tanzanian biodiversity values?*

The NFR network was not formally designed using strategic conservation planning, but was based on a combination of expert knowledge, the framework of ecoregions, and picking the ‘best’ remaining sites owned by the current management body – Tanzania Forest Service (formerly Forestry and Bee-keeping Division of the Ministry of Natural Resources and Tourism). The first designated NFR sites, in the Eastern Arc Mountains or Coastal Forests ecoregions, have higher biodiversity values than more recently declared sites. These first declared regions were known to have globally important biodiversity values [23, 24, 10] and have received more research funding and survey efforts [25, 10] and were thus the first to be protected, with other sites in other ecoregions following as resources/data became available. Many of the newer NFRs have received little or no biodiversity survey attention, so we expect their known values to increase (see e.g., Minziro NFR, [26]). Recently designated sites do contain valuable biodiversity, and contribute to improved biodiversity values within the reserve network (Figures 2: B, C, D). The network of reserves now covers most of the endemic and threatened vertebrate species in Tanzania, however, not their full ranges. The most important sites in terms of biodiversity value also show slightly higher METT scores (Figure 3: B), even though the increase is small.

*What gaps remain in the NFR network?*

As more reserves have been added to the NFR network, the proportion of gap species has decreased for all species groups (Figure 5). This is especially true for endemic mammal and bird species, which respectively have 5% and 0% of gap species remaining in the 2021 network. However, the proportion of poorly covered species increased for some groups such as birds, where more than 30% of species were poorly covered by all the NFRs compared to 26% in only the Amani NFR (Figure 5). Due to the relatively small size of the NFRs and the large range of some Tanzanian endemic species, NFRs could not adequately cover the ranges of all the endemic species in the country. To achieve a larger coverage of the ranges of these species other terrestrial protected habitats have to be included (Figure 7) [27].

The largest number of gap species in the NFR network were from coastal forests (Figures 6 and 7). Even after adding all the types of protected areas in the country, the gap in the south-eastern region of the country remains. There are few and relatively small forest reserves present in that region. Further assessment is needed to determine whether there are additional reserves to cover the distributions of the gap species in south-east Tanzania. Perhaps one of the small forest reserves, such as Rondo or Matapwa Forest Reserve, could be expanded to cover a part of the ranges of gap species, or a new type (mixed forest-woodland habitat) of reserve could be implemented.

*Assessment of the management of the Nature Forest Reserves*

The NFR network in Tanzania has expanded over the past 25 years from a single site to 19 sites covering the main forest types in the country. This network is a valuable asset, especially considering that the world-renowned network of national parks in Tanzania mainly protects non-forest or drier forest habitats, with a few notable exceptions that include Udzungwa Mountains, Mahale, and Kilimanjaro national parks. The future of the NFR network depends on the policies and decisions of the Tanzanian government, but also on the ways in which the sites are funded and managed. Development of the network has been funded by a combination of the Tanzanian government (TFS) and donor funding including the GEF, the Eastern Arc Mountains Conservation Endowment Fund, and private donors such as the Aage V. Jensen Foundation, as well as local and international NGOs. This support has been critical to the observed improvement in management effectiveness (METT scores) from 2015 to 2021 (Figure 3A).

The number of tourists visiting the sites increased from 2015 to 2019, but their contribution to management costs remains small (Figure 3C). Tourism has primarily been by visitors from other countries, yet the number of Tanzanian tourists has also recently increased, especially during the COVID-19 pandemic. Older reserves such as Chome, Magamba, and Amani, with better infrastructure, accessibility, and proximity to the northern tourism circuit, had the largest growth in tourism numbers. However, direct income from tourism in the NFRs remained low, ranging from USD 3447 in 2015 to USD 5032 in 2017 to less than USD 5000 in 2019 (Figure 3D). Since 2020, international tourism has been significantly impacted by the COVID-19 pandemic, which financially weakened these reserves as well as other protected areas in the country. There remains potential for improving local tourism to these NFRs and increasing revenue collection, but the potential to pay the full management costs remains low and care must be taken not to develop facilities that remain underused.

Forest disturbance (using the proxy of cut trees and poles) generally declined from 2001 to 2019 in three of the four studied NFRs, suggesting improved management at these sites. However, this assessment is only available for four reserves, and we have no overview of how this condition may have changed in other sites. Anecdotal evidence suggests that some other reserves are experiencing considerable disturbance, including hunting pressure, that is not captured in the available data. This is the case in Uzungwa Scarp NFR, where decades of poor management of the former Forest Reserve have led to a dramatic loss of wildlife [28, 29]. However, since its upgrading to NFR status in 2016, increased management efforts supported by local and international agencies and implemented in close partnership with TFS have led to a considerable improvement [30]. Key strategies have included capacity building of patrol staff through training workshops and provision of patrol gear, engagement of local community members in collaborative forest patrols, use of novel technologies (such as smartphones to collect patrol data and camera traps for surveillance), and regulated access by communities to non-timber forest products such as firewood, medicinal plants, mushrooms, worshipping sites, and the use of trails for traveling across areas. A similar process has recently begun to show positive results in the Kilombero NFR. Hunting pressure and long-term forest encroachment may have exterminated the endemic Udzungwa forest partridge species from the Nyumbanitu forest within the Kilombero NFR since 2016 [31]. The Nyumbanitu forest is one of the only two forest fragments, both in the Kilombero NFR, where the partridge species occurred. However, law enforcement and raising awareness in local villages since August 2021 are slowly improving the conservation status of this and other single-site endemic species.

*Future management and research*

To improve the management of NFRs, we suggest that future management plans should emphasise enforced hunting and logging regulations at all sites, raising awareness of conservation issues in local villages, and providing local people with viable alternatives to activities that impact the forests and their biodiversity. Such plans have been implemented in the Udzungwa Mountains, Here the TFS has signed agreements with local and international NGOs to support capacity building and facilitate wildlife management, including engaging the local community in intelligence gathering and forest patrols. In addition, we recommend that future management plans include an expansion of the current network, incorporating the endemic gap species and poorly covered species as well as other unprotected forest taxa. Management planning should focus on forest extension, creation, and implementation of meaningful Joint Forest Management regimes between TFS and surrounding communities, improvement of forest quality, and more sustainable use of forest resources.

Monitoring the effectiveness of the management of biodiversity at current and future sites is crucial to maintaining the coverage and efficacy of Tanzania’s NFR network. For example, an equal number of surveys for all taxa and protected areas is currently proposed, beginning with NFRs with little or no biodiversity data (e.g., Hassama, Itulu Hill, Mwambesi, Pindiro, and Uzigua NFRs, all virtually unknown in terms of their flora). Additional surveys of species/taxa distribution patterns are needed, and future research is needed to detect range shifts due to global warming [32]. Furthermore, wildlife life history information, species abundance data and human population growth should be added to future analyses [33].

The implementation of an inflation linked financial budget is necessary to afford new equipment, facilities and infrastructure, and to maintain patrols in the NFR network. To increase the budget for reserve management, we suggest following potential revenue streams: capturing revenue from ecosystem services (such as water catchment and carbon stocks); soliciting additional investment or donations to …, and increasing the revenue generated from tourism [10]. Furthermore, we recommend that TFS consider further expanding the current network to include the small number of endemic gap and poorly covered species that are outside the current NFR network, but could be included with some management status changes at existing sites.

**Limitations**

The data and analyses used here are subject to several limitations. Firstly, it was difficult to assure the quality of METT scores, except METT Version.4.0, and data on recently declared NFRs were missing. Earlier studies have shown that METT data are valuable but need to be interpreted with care when done using self-assessment [17, 34], especially older METT versions, i.e., METT 1-3. To obtain the best outcome from the METT assessment process, studies suggest that METT assessment is done by a group of people to reduce bias [19, 35). In addition, METT 4 requires that local communities are involved in the assessment.

Secondly, only spatial data on the range of species were used, without life history or species population data being considered. This means that the viability of populations within a NFR or within our broader gap analysis is not examined in the METT process. Species range data may also be biased, especially in areas situated farther from protected areas that may have been poorly sampled [27]. Furthermore, defining areas of current species occurrence may be misleading and causing ineffective protection due to the discontinuous distribution of species due to climate change [32].

Thirdly, ecological data on current local threats affecting nature reserves and their taxa were not available in standardized form across the network, with only partially complete data available up to 2019. The COVID-19 pandemic will also have impacted the management and functioning of NFRs in Tanzania, but updated data are not available.

**Acknowledgements**

We thank the many staff of the Tanzania Forest Service and key donors, including the Global Environment Facility (via UNDP and the World Bank), the Critical Ecosystem Partnership Fund, European Union, Governments of Norway, Finland and Denmark, and facilitation by several NGOs: WWF, Tanzania Forest Conservation Group, CARE International, IUCN, WCS, BirdLife International, and African Wildlife Foundation.

**References**

1. Dudley, N. (2008). Guidelines for Applying Protected Area Management Categories. *Research Gate*, DOI:10.2305/IUCN.CH.2008.PAPS.2.en
2. Engh, V. (2011). Integrated Conservation and Development Projects and Efforts Around Amani Nature Reserve, Tanzania, And Their Effects on Livelihoods And Forest Conservation. *Noragric, Department of International Environment and Development Studies*.
3. Burgess, N.D. & Clarke, G.P. (eds) (1990) The Coastal Forests of Eastern Africa. IUCN Forest Conservation Programme, Gland, Switzerland, and Cambridge, UK.
4. Burgess, N.D., Butynski, T.M., Cordeiro, N.J., Doggart, N., Fjeldså, J., Howell, K. et al. (2007). The biological importance of the Eastern Arc mountains of Tanzania and Kenya. *Biological Conservation*, 134: 209 –231.
5. Burgess, N.D., Loucks, C., Stolton, S., Dudley, N. (2007). The potential of forest reserves for augmenting the protected area network in Africa. *Oryx*, 41(2), 151- 159, doi: 10.1017/S0030605307001895
6. United Republic of Tanzania, Ministry of Natural Resources and Tourism (2010). Nomination of properties for inclusion of the World Heritage List Serial Nomination: Eastern Arc Mountains Forests of Tanzania. *United Nations Educational, Scientific and Cultural Organization, Convention Concerning the Protection of the World Cultural and Natural Heritage*. Retrieved from <http://www.whs.tfcg.org/docs/E_Arc_Mountains_World_Heritage_Nomination_100127_FINAL.pdf>
7. URT (UNITED REPUBLIC OF TANZANIA) (1998). National Forest Policy. Ministry of Natural Resources and Tourism, Forestry and Beekeeping Division, Dar es Salaam, Tanzania.
8. URT (UNITED REPUBLIC OF TANZANIA) (2002) The Forest Act. Ministry of Natural Resources and Tourism, Dar es Salaam, Tanzania.
9. Platts, P.J., Burgess, N.D., Gereau, R.E., Lovett, J.C., Marshall, A.R., McClean, C.J. et al. 2011. Delimiting tropical mountain ecoregions for conservation. *Environmental Conservation*, 38: 312–324.
10. Rovero, F., Menegon, M., Fjeldså, J., Collett, L., Doggart, N., Leonard, C. et al. (2014). Targeted vertebrate surveys enhance the faunal importance and improve explanatory models within the Eastern Arc Mountains of Kenya and Tanzania. *Diversity and Distributions*, 20: 1438-1449, DOI: 10.1111/ddi. 12246
11. Rovero, F., Owen, N., Jones, T., Canteri, E., Iemma, A., Tattoni, C. (2016). Camera trapping surveys of forest mammal communities in the Eastern Arc Mountains reveal generalized habitat and human disturbance responses. *Biodiversity Conservation*, 26: 1103–1119, DOI, 10.1007/s10531-016-1288-2
12. Rovero, F., Shinyambala, S., Perkin, A., Greco, I. (2019). Minziro Nature Reserve Biodiversity Surveys 2018. FigshareFigshare.com/articles/Minziro_Nature_Reserve_Biodiversity_surveys_2018/10283822/1 [accessed 8 July 2020].
13. Burgess, N.D. & Clarke, G.P. (eds.) (2000) Coastal forests of eastern Africa. *IUCN Forest Conservation Programme*. Cambridge University Press, Cambridge.
14. BirdLife International and Handbook of the Birds of the World (2019) Bird species distribution maps of the world. Version 2019.1. Available at <http://datazone.birdlife.org/species/requestdis>.
15. IUCN (2020). The IUCN Red List of Threatened Species. Version 2020-3. Available at https://www.iucnredlist.org. Downloaded on 27/10/2020.
16. Roll, U., Feldman, A., Novosolov, M., Allison, Allen., Bauer, A.M., Bernard, R. et al. (2017). The global distribution of tetrapods reveals a need for targeted reptile conservation. *Nature Ecology & Evolution*, 1, 1677–1682, DOI: 10.1038/s41559-017-0332-2
17. Stolton, S., Dudley, N., Belokurov, A., Deguignet, M., Burgess, N.D., Hockings, M. et al. (2019). Lessons learned from 18 years of implementing the management effectiveness tracking tool (METT): A prospective from the METT developers and implementers. *Parks,* 10.2305, DOI: 10.2305/IUCN.CH.2019.PARKS-25-2SS.en
18. Knights, K., Cuadros, I., Zamora, C., Coad, L., Leverington, F., O’Connor, B. et al. (2014). A preliminary assessment of protected area management within the WWF ‘Coastal East Africa’ priority place, eastern Africa. *Parks*, 20.2: 78-88.
19. Coad,L., Leverington, F., Knights, K., Geldmann, J., Eassom, A., Kapos, V. et al. (2015). Measuring impact of protected area management interventions: current and future use of the Global Database of Protected Area Management Effectiveness. *Philosophical Transactions of the Royal Society B Biological Sciences*, 370 : 20140281. http://dx.doi.org/10.1098/rstb.2014.0281
20. UNDP GEF (2014). Project document. PIMS5106. Enhancing the Forest Nature Reserves Network in Tanzania.
21. R Core Team (2021). R: A language and environment for statistical computing. R Foundation for Statistical Computing, Vienna, Austria.
22. Ahrends, A., Bulling, M.T., Platts, P.J., Swetnam, R.D., Ryan, C.M., Doggart, N. et al. (2020). Detecting and prediction forest degradation: A comparison of ground surveys and remote sensing in Tanzanian forests. *Plants People Planet*, 2021; 3:268-281
23. Burgess, N.D., Clarke, G.P., Rodgers, W.A. (2008). Coastal Forests of eastern Africa: status, species endemism and its possible causes. *Biological Journal of the Linnean Society,* 64: 337-367.
24. Burgess, N.D., Malugu, I., Sumbi, P. Kashindye, A., Kijazi, A., Tabor, K. et al. (2017). Two decades of change in state, pressure, and conservation responses in the coastal forest biodiversity hotspot of Tanzania. *Oryx*, 51(1), 77-86, doi:10.1017/S003060531500099X
25. Ahrends, A., Burgess, N.D., Gereau, R.E. Marchant, R., Bulling, M.T., Lovett, J.C. et al. (2011). Funding begets biodiversity. *Biodiversity Research*, DOI: 10.1111/j.1472-4642.2010. 00737.x
26. Greco, I and Rovero, F. (2021). The African golden cat Caracal aurata in Tanzania: First record and vulnerability assessment. *Oryx*, 55: 212:215215. doi:10.1017/S003060532000040X
27. Klein, C.J., Brown, C.J., Halpern, B.S., Segan, D.B., McGowan, J., Beger, M. et al. (2015). Shortfalls in the global protected area network at representing marine biodiversity*. Scientific Reports*, 5:17539, DOI: 10.1038/srep17539
28. Rovero, F., Arafat, S.A., Kitegile, A., Nielsen, M.R. (2012). Hunting or habitat degradation? Decline of primate populations in Udzungwa Mountains Tanzania: An analysis of threats. *Biological Conservation*, 146: 89-96.
29. Oberosler, V., Tenan, S., Zipkin, E.F., Rovero, F. (2020). Poor management in protected areas is associated with lowered tropical mammal diversity. *Animal Conservation*, 23: 171-181, doi:10.1111/acv.12525
30. Southern Tanzania Elephant Program, (2021.). STEP 2021 Annual Report. <https://stzelephants.or.tz/download/annual_reports/STEP_2021_AnnualReport_Digital.pdf>
31. Dinesen, L., Jensen, F.P., Sonne, J., Lenvinsky, I., Mulungu, E. (2021). Status and habitat description of the globally threatened Udzungwa Forest Partridge Xenoperdix udzungwensis thirty years after discovery. *Bird Conservation International*. <https://doi.org/10.1017/S0959270921000319>
32. Prendergast, J.R., Quinn, R.M., Lawton, J.H. (1998). The Gaps between Theory and Practice in Selecting Nature Reserves. *Conservation Biology*, Pages 484-492, Volume 13, No. 3, June 1999
33. Lomolino, M.V. (1994). An Evaluation of Alternative Strategies For Building Networks Of Nature Reserves. *Biological Conservation*, 69 (1994), 243-249
34. Geldmann, J., Coad, L., Barnes, M.D., Craigie, I.D., Hockings, M., Knights, K. et al. (2015). Changes in protected area management effectiveness over time: A global analysis. *Biological Conservation*, 191, (2015), 692-699, <http://dx.doi.org/10.1016/j.biocon.2015.08.029>
35. Geldmann, J., Coad, L., Barnes, M, D. Craigie, I.D., Woodley, S., Balmford, A. et al. (2017). A global analysis of management capacity and ecological outcomes in terrestrial protected areas. *Conservation Letters*, DOI: 10.1111/conl.12434. <https://doi.org/10.1111/conl.12434>

**Supporting information**

**Table 3:** Basic attributes of 19 declared and three proposed Tanzanian Forest Nature Reserves

| **NFR NAME** | **ZONE** | **REGION** | **DISTRICT** | **AREA (Ha)** | **YEAR GAZETTED** | **TOTAL TANZANIAN ENDEMIC PLANT TAXA** | **TOTAL TANZANIAN ENDEMIC VERTEBRATE SPECIES** | **TOTAL NUMBER OF SINGLE SITE ENDEMIC VERTEBRATE SPECIES** | **TOTAL THREATENED TAXA** |
| --- | --- | --- | --- | --- | --- | --- | --- | --- | --- |
| Amani | North | Tanga | Korogwe & Muheza | 8,380 | 1997 | 122 | 53 | 10 | 162 |
| Chome | North | Kilimanjaro | Same | 14,283 | 2016 | 48 | 14 | 8 | 87 |
| Itulu hills | West | Tabora | Sikonge | 388,512 | 2019 | 0 | 2 | 0 | 24 |
| Kalambo | SH | Rukwa | Kalambo | 41,958 | 2019 | 1 | 0 | 0 | 19 |
| Kilombero | Southern Highlands | Iringa & Morogoro | Kilolo & Kilombero | 134,511 | 2007 | 94 | 69 | 1 | 151 |
| Magamba | North | Tanga | Lushoto | 9,284 | 2016 | 34 | 32 | 4 | 92 |
| Magombera | East | Morogoro | Kilombero | 2,615.32 | 2019 | 9 | 4 | 0 | 39 |
| Minziro | Lake | Kagera | Misenyi | 25,717 | 2016 | 0 | 0 | 1 | 28 |
| Mkingu | East | Morogoro | Mvomero | 26,433 | 2016 | 77 | 51 | 15 | 114 |
| Mount Hanang | Central | Manyara | Hanang | 5,871 | 2016 | 8 | 5 | 1 | 29 |
| Rungwe | SH | Mbeya | Mbeya Rural & Rungwe | 13,652 | 2009 | 39 | 61 | 3 | 64 |
| Mwambesi | South | Ruvuma | Tunduru | 112,901 | 2019 | 0 | 0 | 0 | 15 |
| Nilo | North | Tanga | Korogwe & Muheza | 6,025 | 2007 | 33 | 51 | 0 | 97 |
| Pindiro | South | Lindi | Kilwa | 12,249 | 2019 | 1 | 0 | 0 | 21 |
| Rondo | South | Lindi | Lindi Rural & Ruangwa | 11,742 | 2007 | 26 | 10 | 3 | 80 |
| Uluguru | East | Morogoro | Morogoro Rural, Morogoro Urban & Mvomero | 24,115 | 2008 | 198 | 51 | 14 | 224 |
| Uzungwa Scarp | SH | Iringa & Morogoro | Kilombero, Kilolo & Mufindi | 32,763 | 2016 | 58 | 72 | 5 | 133 |
| Pugu-Kazimzumbwi | East | Dar es Salaam & Pwani | Ilala, Kinondoni, Kisarawe & Mkuranga | 8,965.30 | 2020 | 27 | 5 | 0 | 72 |
| Uzigua | East | Pwani & Tanga | Chalinze, Handeni & Kilindi | 27654.58 | 2020 | 0 | 0 | 0 | 23 |
| Essimingor | North | Arusha | Monduli | 6070 | PROPOSED | 1 | 0 | 0 | 35 |
| Hassama Hill | Central | Manyara | Babati & Mbulu | 4901 | PROPOSED | 0 | 0 | 0 | 27 |
| Nou | Central | Manyara | Babati & Mbulu | 28936 | PROPOSED | 2 | 2 | 0 | 28 |
