# Supplementary material for: Nature Forest Reserves in Tanzania and their importance for conservation
Source: PLoS One. 2024 Feb 5;19(2):e0281408. doi: 10.1371/journal.pone.0281408 (PMC10843475; doi:10.1371/journal.pone.0281408)
Supplement: Attachment — Submitted filename: Response to reviewers -rebuttal Letter.docx [file pone.0281408.s005.docx]

Rebuttal Letter (the line number correspond to the revised manuscript)

First, many thanks for your review of our paper. We have taken the time to go back to colleagues in Tanzania and update all the base data we have used for the paper (number of reserves, tourism changes over time, any other changes we can add to bring the paper 100% up to date).

As the raw data have in some cases changed due to new information being received from the government in Tanzania, we have re-made the location figure and the graphs. We have also re-run the various statistical tests according to the recommendations made by the reviewer.

As new data have been received from Tanzania, we have increased the number of Tanzanian authors on the paper.

We have also taken the opportunity to clarify the data used, methods behind the analyses, aim of the analyses, and results from the analyses. Our main goal in the analysis has been to both show the values of the existing nature reserves network and how this has changed over time, and highlighting the remaining areas in Tanzania where endemic species are not included in nature reserves, or within any other kind of reserve. This sequential gap analysis approach is useful for conservation planning purposes and does highlight the remaining areas that might need to be conserved to cover all the endemic species where we have distribution data.

Response to major question raised by reviewer:

*This is a useful and encouraging review of the conservation status of forest reserves and their vertebrate wildlife in Tanzania. The manuscript reads like a report rather than a critical review and conclusions are framed as suggestions*.

**Response**: We have re-drafted the text of the paper and tried to sharpen it as a scientific paper.

*A more decisive statement of priorities to ensure positive outcomes for the NFR network is needed. The statistical analyses rely on non-parametric correlation, which is incorrect because time is incorrectly used as the independent variable when, in fact, it is a factor (i.e., the year in which a METT assessment was done). ANOVA or its non-parametric equivalents (Kruskal-Wallis; Friedmann’s test) are more appropriate but see below for comments about using a systematic conservation planning approach.*

**Response**: We have re-made all the statistics in the paper using suggested approaches

*Stacked bar-graphs are difficult to interpret in some cases and another graphic method may be more useful. Maps lack legends and scales.*

**Response**: We have reviewed the figures and where possible we have changed the format, and we have reviewed the legends and labelling and tried to improve this throughout

*The writing is reasonable but can be finessed and improved. I have done an extensive edit of the manuscript using tracked changes and attach it to my review.*

**Response:** We have accepted the proposed edits with thanks and sought to further improve the language throughout the paper

*That said, I think this is a useful paper and could be published but falls way short of its potential. I would have liked to see more critical assessment and encourage the authors to consider upgrading their analyses.*

*This can be achieved by using well established iterative algorithms that allow for systematic identification of priority reserves based on species rarity and complementarity. The latter would up-date the research to contemporary practices. See the following references:*

**Response**: We are aware of the systematic conservation planning literature and several of the authors have used these approaches at various geographical scales and we have published papers using these approaches over the past 25 years. We believe our analytical approach is sound because species endemic to Tanzania with small ranges would drive complementarity analyses and have sought to greatly clarify what we have done in light of this comment.

**Response:** We have clarified in the paper that we have done 2 main analyses of conservation priority and we have re-run the analyses using the latest data on the number of Nature Forest Reserves and other reserves in Tanzania.

1) The first analysis uses the species lists available from the Nature Forest Reserves themselves. This is a new database, and the plants database took over 3 years to create by an expert. We have used these data to rank the values of the reserves.
2) The second analysis uses spatial data for all vertebrates in Tanzania, with an emphasis on endemic and threatened species. For these data we apply a sequential approach to determining the species already present within the existing Nature Forest Reserves, and then within other kinds of reserves in the country. This approach provides a clear set of priorities to TFS on the remaining species that occur outside the protected areas. We feel this is more operationally useful than a systematic conservation planning approach that does not necessarily consider the existing reserves and their sequence of creation and success.

*Without analyses that provide insights to which reserves are a priority for conservation, the conservation conclusions are platitudinous and not implementable.*

*The suggestion that “…we recommend that TFS consider further expanding the current network to include the small number of endemic gap and poorly covered species that are outside the current NFR network but could be included with some management status changes at existing sites.” is the sort of recommendation I was expecting and could be elicited by a systematic conservation planning approach.*

**Response**: We have sought to clarify our approach. We use distribution data for all Tanzania endemic species in our analysis and have undertaken a stepwise analysis where we excluded from the analysis all species covered by the Nature Forest Reserves, and then all other kinds of reserves, to show the remaining gaps. As our paper was focused on the existing network of Nature Reserves, and their development over time, we sought to provide an assessment of the ‘success’ of this network and provide guidance to TFS who are seeking to further expand the network to critical areas. We have clarified the approach taken. And we have provided clearer guidance to TFS.

Through the updating process a series of TFS (Tanzania government) authors have asked to be included in the paper, which we think is testimony to the success of our approach in terms of delivering an impact on reserve creation and management in Tanzania.

**Line 39** – Comment “Might be useful to say what the response variable used in the time series is- i.e., how is effectiveness measured?”. **Response** “We added the response variable used: the management effectiveness tracking tool (METT). How the management effectiveness is measured is explained in the method part”.

**Line 44** – Comment “Will further NFRs be declared and given the raft of challenges, which suggest that local communities are under pressure, will human-wildlife interactions receive more attention as improving the well-being of local people. In other words, are their contingency plans to ensure that the NFRs are sustainable within the context of the needs of local society. Just a suggestion – otherwise you leave the reader with a catalogue of challenges and no hint of possible solution via integrated management.” **Response** “We added a sentence that included different solutions required to create a network of economically self-sustaining NFRs, able to conserve critical biodiversity values and take into account the needs of local society”.

**Line 96** – Comment “Remove global perspective from map; insert lat -long grid on x and y axes, remove fuzzy orange border to colored areas.” (From Figure 1). **Response** “The modifications were done on Figure 1”.

**Line 212 (Figure 2C)** – Comment “Is it possible that the current NFR coverage has reached threatened species saturation?”. **Response** “We think that the current NFR coverage is moving towards saturation. However, our analysis does show that there are some more species endemic to Tanzania that could be captured in the enhanced network, therefore, the saturation is not achieved yet”.

**Lines 208 and 209 –** Comment “Change rho to this symbol throughout”. **Response** “The modification has been completed”.

**Line 209 (the part is now removed)** **–** Comment “Spurious correlation as it lacks a question – why would you expect a relationship?”. **Response** “Indeed, we wanted to test different parameters together, but this one was a mistake. Therefore, the specific sentence was removed from the updated manuscript.”

**Line 211 –** Comment “Difficult to see the differences in the stacked bar charts in Figure 2A– is there another way to display these data?”. **Response** “Unfortunately we chose the stacked bar, as it is the easiest way to display our data with the 5 different taxa, but to make it easier to see the differences, we decided to change the color of figure 2”.

**Line 274–** Comment “I am puzzled by this correlation as it seems to lack a specific prediction and is meaningless, especially as the explanatory variables are in fact factors – use no parametric ANOVA? Remember that the correlation metric is strictly linear Perhaps phrase it differently “Management effectiveness has improved greatly in recent years””. **Response** “We wanted to look for a possible correlation between the date of establishment of a reserve and its management effectiveness value: our hypothesis was the following: the oldest the date of establishment of a reserve was the highest the management effectiveness will be. However, the results were not significant. But we phrased it as suggested “Management effectiveness has improved in recent years”.

**Line 274 until** **line 279 –** Comment “Another spurious correlation – I wonder if this analysis and the previous correlations wouldn’t be better done using one-way Kruskal -Wallis non-parametric ANOVA or Friedmann's test which compares the medians of three or more dependent groups and is the nonparametric equivalent of the two - way ANOVA”. **Response** “For this analysis, we wanted to check if there was a possible link between the reserves with more endemic taxa and the management effectiveness: we expected that reserves with a higher management effectiveness will have better management plans, and therefore might be able to conserve more endemic species. However, we decided to not use the one-way Kruskal -Wallis non-parametric ANOVA or Friedmann's test because the total number of endemic taxa have been calculated only one time for each reserve, in comparison to the METT scores that have been calculated several times for the four years studied. Furthermore, the two groups (number of endemic taxa and METT scores) have a different unit. Therefore, we decided to delete the figure 6B and the associated sentence in the text, as indeed the two variables seems to be incompatible to be tested significantly”.

For the previous correlation however, we used the Friedman test: testing the significant differences observed between the median of the METT scores in 2015, 2017, 2019 and 2021. Furthermore, we also used the post hoc test (pairwise Wilcoxon rank sum test) to test between which years, the statistical difference was the most important”.

**Line 136** – Comment “Not sure what this means – also belongs in methods”. **Response** “This means that the currency of the income was in US dollars. This sentence was added in the method: line 136”.

**Line 283 until line 287** – Comment “Analyses are not correct for the question being posed. In this case, average annual income has decreased notably from 2017 to 2019 Friedman’s test”. **Response** “We used the Friedman test as suggested to test the significant differences observed between the medians of the income generated through the years studied. Furthermore, as above, we also used the post hoc test (pairwise Wilcoxon rank sum test) to test between which years, the statistical difference was the most important”.

**Line 290** – Comment “‘small’ surely?”. **Response** “We changed this to “office equipment”.

**Line 297** – Comment “check”. **Response** “We changed this to “office equipment”.

**Line 303** – Comment “significantly?”. **Response** “Unfortunately, none of these results were significant”.

**Line 303** – Comment “deleted interpretation because I think it is wrong given that disturbance has decreased”. **Response** “Interpretation was removed”.

**Line 305** – Comment “Change y-axis label to “Mean no. of trees per ha”. Drop “average” and “live”. **Response** “This was changed in the new updated figure”.

**Line 226** – Comment “Can this sentence be more specific i.e., what proportion. Also, clarify the point you are trying to make here. It is a confusing sentence to interpret”. **Response** “We added the proportion of Tanzanian endemic amphibian, and we modified the sentence stating that more than 25% of the 65 amphibian’s endemic species are partially covered by the reserves”.

**Line 231** – Comment “Insert legend at the bottom of the figures”. **Response** “We inserted a legend at the bottom of the figure 3”.

**Line 247 –** Comment “Insert this information on the figures as headings and legends. It is very difficult for the reader to compare times and taxa without the latter”. **Response** “This part was inserted in the figure 4 as headings and legends”.

**Line 261** **–** Comment “Insert this information into a legend”. **Response** “This information was inserted into a legend in Figure 5”.

**Line 315** **–** Comment “How is this possible if the Amani reserve is part of the overall network?”. **Response** “Indeed, this comparison did not make any sense. It was removed from the updated manuscript”.

**Line 383–** Comment “check”. **Response** “After talking to my colleagues, the best solution would be to expand one of the small forest reserves already established instead of implementing a new type of reserve. So, this sentence was removed from the revised manuscript”.

**Line 343** – Comment “Expand”. **Response** “This was expanded in the updated manuscript”.

**Line 363** **–** Comment “Although this ‘improvement’ could be due to there being fewer suitable trees and poles to harvest”. **Response** “Indeed. To check this, we need to look if the density of live trees and lives poles has changed: if live trees have declined, then this could be possible and if live poles have increased, this could be also possible (as the poles are coming back in areas where the trees have been cut). We added a new sentence taking this possibility into account”.

**Lines 391 until 393 –** Comment “A citation would be useful as it will allow readers to look at how this was done”. **Response** “This part was removed, as we did not want to give an example of improvement happening in a specific reserve, but we wanted to give recommendations for all the NFRs. A new paragraph highlighting our recommendations for a more effective coverage and protection of endemic gap species, as well as management improvement was added”.

**Lines 399 –** Comment “citation” (on the implementation of Joint Forest Management). **Response** “The part on Joint Forest Management was removed because Joint Forest Management was observed in other type of protected areas but not in NFRs, therefore, we do not have any concretes examples if this type of management could actually work in a NFR”.

**Line 403 –** Comment “Perhaps some citations to REDD+ schemes may be useful here”. **Response** “We added papers on: (i) REDD + progress and challenges in Tanzania (ii) a document published by the Tanzania government and his project to make Tanzania REDD+ ready for the implementation of Paris Agreement and to contributes to the country reduction of carbon emissions (iii) a local NGO in the country called “carbon Tanzania”, developing forest conservation projects in collaboration with forest communities to generate forest carbon credits and allows forest communities to earn revenues in return for measurable and verifiable forest protection activities: citations 37, 38 and 39”.

**Line 403 –** Comment “Investment in what exactly? Investment only occurs where there is a tangible return – without identifying what investments this is nothing more than a wish list”. **Response** “Investment or donations coming from philanthropic donors’ sources to increase the budget for reserve management”.

**Line 176 –** Comment “I recommend that this whole section be incorporated into the methods section”. **Response** “This section was added in the methods section”.
